# A plea for avoiding systematic intubation in severely hypoxemic patients with COVID-19-associated respiratory failure

**DOI:** 10.1186/s13054-020-03063-6

**Published:** 2020-06-12

**Authors:** Eduardo Villarreal-Fernandez, Ravi Patel, Reshma Golamari, Muhammad Khalid, Ami DeWaters, Philippe Haouzi

**Affiliations:** 1 Division of Pulmonary and Critical Care Medicine, Department of Medicine, Penn State Health, Pennsylvania State University, College of Medicine, 500 University Drive, Hershey, PA 17033 USA; 2 Division of Hospital Medicine, Penn State Health, Pennsylvania State University, College of Medicine, Hershey, PA USA

To the Editor,

In early February 2020, Yang et al. [1] reported an alarming high mortality rate in patients with COVID-19-associated acute respiratory failure requiring mechanical ventilatory support. Such a dreadful outcome was regarded as the fundamental tenet dictating our strategy to treat patients with COVID-19 acute respiratory failure. Two essential recommendations were offered to the medical community in keeping with these first reports: (1) early intubation of hypoxemic patients [2]. Indeed, since a profound hypoxemia appears to be the hallmark of COVID-19-associated pneumonia, the initial consensus [2] was to start invasive mechanical ventilation as soon as possible due to the overwhelming number of patients in respiratory failure presenting at the same time in a hospital and to prevent the risk of hypoxic cardiac arrest; (2) avoidance of high-flow nasal cannula (HFNC) to reduce respiratory droplet aerosolization for healthcare workers [3] in what was seen as “inevitable” intubations.

During the very initial weeks preceding the anticipated surge in central Pennsylvania, ten patients with confirmed infection by SARS-CoV-2, who had extremely high oxygen requirement, were admitted in our institution (Harrisburg/Hershey region): all patients required a high flow of oxygen by nasal cannula (NC) or via non-rebreather (NRB) (Fig. 1) with documented episodes of SpO_2_ < 90%. The first four patients underwent endotracheal intubation by day 2 of hospital admission without a trial of high flow nasal cannula (HFNC) or non-invasive ventilation (NIV), following the recommendations for early intubation [2]. However, we reconsidered in other patients the rationale behind these early intubations and revisited the initial proposal of avoiding high flow oxygen in hypoxemic patients. In addition, we felt that the actual consequences of aerosolization posed by HFNC and NIV [4] remain quite hypothetical as reported in H1N1 pneumonia [5]. Our main concern was that a systematic intubation of every hypoxemic patient may prove to be untenable, facing a limitation of capacity and resources of intensive care units (ICU) to safely maintain a high number of patients on mechanical ventilation during the expected surge. We therefore selected a different strategy in the following six patients whose initial oxygen requirement was in the same range as the patients who were intubated by day 2. Empiric limit of hypoxic events ~ 88% was considered acceptable as long as the SpO_2_ was maintained at or above this level during most of the day and could be improved by self-prone positioning. This strategy was adopted in the absence of preexisting chronic respiratory failure, morbid obesity, concurrent clinical signs of respiratory distress, hypercapnia, alteration in hemodynamics, or lactic acidosis. We used a flow of oxygen up to 6 L/min NC and HFNC whenever higher FiO_2_ was needed (Fig. 1). Out of these six patients, two required invasive mechanical ventilation after failing HFNC: one patient developed respiratory fatigue and required intubation at day 3, while the second patient had intolerance to NIV and self-prone positioning with an episode of emesis that led to intubation on day 6. The non-intubated patients were instructed to rest in a prone position as much as feasible. After an initial increase in oxygen requirement through day 6, patients in this group were all able to be discharged at a time when most of the early-intubated patients were still mechanically ventilated (Fig. 1). Strikingly, this occurred despite similar initial oxygen requirements.

**Fig. 1:**
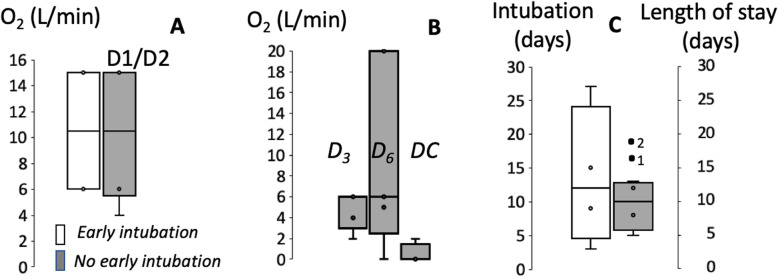
**a** Oxygen flow requirement (median and range) during the first 48 h of admission in the group of patients who were intubated within the first 2 days based on the level of hypoxemia (4 patients, white bars) and those who were not immediately intubated (6 patients, grey bars). **b** Evolution of O_2_ requirement in patients that were not intubated initially and did not require any mechanical ventilation thereafter (4 out of 6). Note that O_2_ requirement increased over the first week in this group and required the use of HFNC in many patients. They all recovered within 12 days. **c** Duration of intubation in the “early-intubation” group and length of stay in the patients that were not initially intubated. The 2 patients whose intubation was delayed are displayed as (1) and (2). Patient 1 was extubated at day 16, while the second patient was still intubated at day 19 (when this report was submitted)

In summary, avoiding endotracheal intubation is possible in significantly hypoxemic COVID-19 patients. The rationale that led to the practice patterns suggested in earlier reports must be reevaluated, and a controlled graduated method of escalating oxygen therapy, based on individual clinical judgment, in otherwise non-distressed patients should be instituted as much as possible. Such an approach remains to be standardized.

## Data Availability

All data generated or analyzed during this study are included in this published article.
